# Deep learning with diffusion MRI as in vivo microscope reveals sex-related differences in human white matter microstructure

**DOI:** 10.1038/s41598-024-60340-y

**Published:** 2024-05-14

**Authors:** Junbo Chen, Vara Lakshmi Bayanagari, Sohae Chung, Yao Wang, Yvonne W. Lui

**Affiliations:** 1 Department of Electrical and Computer Engineering, New York University Tandon School of Engineering, 370 Jay Street, 9th Floor, Brooklyn, NY 11201 USA; 2 Center for Advanced Imaging Innovation and Research (CAI2R), Department of Radiology, New York University Grossman School of Medicine, New York, NY USA; 3 Bernard and Irene Schwartz Center for Biomedical Imaging, Department of Radiology, New York University Grossman School of Medicine, New York, NY USA; 4 Department of Biomedical Engineering, New York University Tandon School of Engineering, Brooklyn, NY USA

**Keywords:** Magnetic resonance imaging, Diffusion tensor imaging

## Abstract

Biological sex is a crucial variable in neuroscience studies where sex differences have been documented across cognitive functions and neuropsychiatric disorders. While gross statistical differences have been previously documented in macroscopic brain structure such as cortical thickness or region size, less is understood about sex-related cellular-level microstructural differences which could provide insight into brain health and disease. Studying these microstructural differences between men and women paves the way for understanding brain disorders and diseases that manifest differently in different sexes. Diffusion MRI is an important in vivo, non-invasive methodology that provides a window into brain tissue microstructure. Our study develops multiple end-to-end classification models that accurately estimates the sex of a subject using volumetric diffusion MRI data and uses these models to identify white matter regions that differ the most between men and women. 471 male and 560 female healthy subjects (age range, 22–37 years) from the Human Connectome Project are included. Fractional anisotropy, mean diffusivity and mean kurtosis are used to capture brain tissue microstructure characteristics. Diffusion parametric maps are registered to a standard template to reduce bias that can arise from macroscopic anatomical differences like brain size and contour. This study employ three major model architectures: 2D convolutional neural networks, 3D convolutional neural networks and Vision Transformer (with self-supervised pretraining). Our results show that all 3 models achieve high sex classification performance (test AUC 0.92–0.98) across all diffusion metrics indicating definitive differences in white matter tissue microstructure between males and females. We further use complementary model architectures to inform about the pattern of detected microstructural differences and the influence of short-range versus long-range interactions. Occlusion analysis together with Wilcoxon signed-rank test is used to determine which white matter regions contribute most to sex classification. The results indicate that sex-related differences manifest in both local features as well as global features / longer-distance interactions of tissue microstructure. Our highly consistent findings across models provides new insight supporting differences between male and female brain cellular-level tissue organization particularly in the central white matter.

## Introduction

Biological sex (throughout this manuscript, sex, male and female refer to biological sex assigned at birth) is a crucial variable in neuroscience research. The National Institutes of Health require all preclinical and human subject studies to account for biological variables including sex in the research plan. Understanding of sex differences is particularly important as it has been shown to relate to a wide range of cognitive functions such as motor cognitive performance^1–3^, nonverbal reasoning^3^, working memory^4–6^ and episodic memory^7–9^. In addition, prevalence of several neurological and neuropsychiatric disorders differs between males and females. For example, autism spectrum disorder and Tourette syndrome are more prevalent in males^10,11^, while disorders such as multiple sclerosis and depression are more prevalent in females^12,13^.

Recent advances in MRI have enabled precise measurement of the brain noninvasively. However, most MRI studies have documented structural differences between sexes in terms of gross brain volume^14,15^ and cortical thickness^16^ though inconsistencies exist between reports throughout individuals^17–19^. Perhaps of greater interest is whether there exist differences in cellular-level organization of the brain between males and females. Better understanding underlying sex differences in brain microstructure would inform how biological sex influences brain health and disease. Indeed, cellular-level microstructure is known to be informative in various brain studies including brain development, aging, and neurological diseases such as demyelination, tumor infiltration, and dementia^20–23^. Some studies have been conducted on ex vivo samples from animal models that look at cellular features such as density of microglia^24,25^; however, a true picture of the brain’s cellular structure in vivo remains elusive and ex vivo studies are limited by fixation and preparation artifacts which invariably alter the cellular matrix.

Diffusion MRI is capable of capturing microscopic features of the brain noninvasively^26^ and is actively being used to study various neurological diseases, ranging from neurodegenerative disorders such as Alzheimer’s dementia^27,28^ and Parkinson’s disease^29^ to autoimmune disorders such as Multiple Sclerosis^30^. Previous studies have found evidence to support the sex differences in diffusion parameters. For example, in different age ranges, analyses have indicated sex differences in various diffusion parameters such as fractional anisotropy (FA)^16,31–36^, orientation dispersion^16,35,36^, mean diffusivity (MD)^32,34,36^, axial diffusivity (AD) and radial diffusivity (RD)^32,36^. The sex differences have been indicated in various white matter regions such as thalamic radiation^16,32^, cerebellar^31^, superior longitudinal fasciculus^31,32^, corpus callosum^31,32,36^, corona radiata^32^. However, of the studies that have been conducted, most rely on conventional statistical analysis methods in various regions-of-interest^16,31,32,35,36^. The results have been criticized as rigorous correction for multiple comparisons seems to diminish the power of such differences, which have raised debate and the need for new approaches to study the sex differences in brain^37^.

Recent advances in deep neural networks provide advanced methods to capture sex differences in brain microstructure. Two recent works using neural networks with handcrafted features from structural connectivity indicated sex-related differences^33,34^; however, the use of complex hand-crafted features has again been criticized by some as cumbersome, adding potential biases, and limited in reproducibility. Besides, as different deep neural networks architectures are highly likely biased toward different types of features^51,54^, it is challenging for studies based on a single model or similar models to capture thorough information. Therefore, inclusion of multiple distinct model architectures is necessary.

In this work, we have designed a comprehensive, rigorous learning-based approach aimed at contributing new evidence and insights of sex differences to the debate on whether there are indeed sex-related differences in the human brain microstructure. We hypothesize that sex differences exist in brain microstructure as various types of features, such as local features and global interactions. We accomplish this by leveraging end-to-end deep neural networks and diffusion MRI able to tap in vivo brain microstructure. The end-to-end design obviates the need for complex, a priori choices for either choosing of ROIs or hand-crafted feature engineering that could bias analysis. We register all subjects’ brains to a standard template space to remove the potential influence of overall brain size and volume. In addition, we explore 3 major, popular network architectures that capture different and complementary information and thus this work does not rely on a single model choice or model type. Finally, we attempt to identify WM areas that contribute most significantly to sex classification, and thereby have the most embedded sex-related differences. Instead of building a sex classifier, the goal of this work is to provide new evidence and insights regarding sex-related differences in brain tissue microstructure.

## Materials and methods

### Study population

The study includes 1031 healthy adult subjects (age range, 22–37 years) from the Human Connectome Project (HCP—Young dataset)^38^, whereby sex labels were collected through self-reporting and no subject was found to have different self-reported sex from genetic sex. Institutional review board approval and participants’ informed consent were obtained at the participating institutions. Demographic details are summarized in Table 1.

**Table 1:** Study cohort.

	Male	Female
Number of subjects	471	560
Age range: number of subjects		
22–25	138	79
26–30	205	249
31–37	128	232

### Diffusion MRI

Diffusion MR images were collected on a 3T scanner (Connectome Skyra, Siemens Medical Solutions, Erlangen, Germany) and preprocessed as per HCP protocol^38,39^. In brief, diffusion imaging was performed with the following parameters: 3 b-values (1000, 2000, 3000 s/mm^2^), 90 diffusion orientations per shell, 18 b_0_ (b-value = 0) images, 1.25 mm isotropic image resolution, field of view = 210 mm, number of slices = 111, TR/TE = 5520/89.5 ms, each scan was repeated along 2 phase encoding directions (RL/LR), details can be found in HCP dataset^38^. The diffusion data was preprocessed by HCP for correction of artifacts like motion and eddy-currents artifacts^39^. We use a in-house image processing tool to generate diffusion metrics^40^, including fractional anisotropy (FA), mean diffusivity (MD) and mean kurtosis (MK) to assess white matter microstructure. FA and MD are included because they are the two most commonly used diffusion metrics for characterization of tissue microstructure in many studies^41^. Of note, FA measures directionality of water movement in brain tissue, known to be sensitive to microstructures such as axons and myelin^42^; and MD measures mean water diffusivity, sensitive to characteristics like cellularity^43^. Here, we also include MK derived from diffusion kurtosis imaging (DKI) to compactly represent non-Gaussian behavior of water molecules as a measure of tissue complexity^44^. All metrics are registered to the FA template in the MNI space^45^ using FMRIB Software Library (FSL)^46^ so as to remove effects of any macroscopic anatomical differences such as size and contour of the brain itself.

### End-to-end classification models

This study employs three major model architectures: 2D convolutional neural network (CNN)^47^, 3D CNN^48–50^, and 3D vision transformer (ViT)^51,52^. We choose these end-to-end deep networks that act on the entire image volume to avoid any reliance on hand-crafted features and/or complicated feature engineering. In general, CNN and ViT show state-of-the-art performances broadly across image classification tasks. The two architectures have their own strengths and may be complementary: CNN has inductive bias by design such as locality and translation equivalence/invariance (with/without pooling), making such a model generally more sample-efficient and easier in theory to capture local features of an image or volume^53^. While ViT lacks the inductive bias from convolutional layers rendering them somewhat more data-hungry, ViT has strengths that CNNs lack in being able to capture long-range interactions and more global features present in an image or imaging volume^54,51^, which could be important for capturing potential sex differences exist as long-range interactions. Although 3D CNN may be an intuitive choice of architecture to handle a 3D imaging volume, a 3D CNN requires more parameters and more training samples compared with a 2D CNN. Thus, we also test the performance of a 2D CNN with a lighter feature extraction backbone and greater training efficiency.

#### 2D convolutional neural network

**Figure 1:**
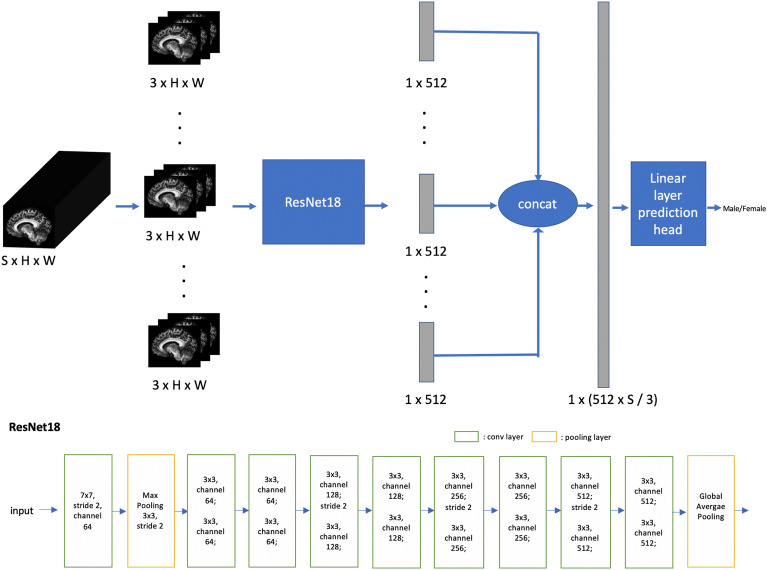
Our 2D CNN model. In the top of the figure, the imaging volume is divided into subvolumes, and a shared ResNet18 is applied to extract 512 features from each subvolume. The features are concatenated and fed to a linear layer for the final prediction. The bottom of the figure shows the architecture of ResNet18 (residual connection, ReLU activation, batch normalization are omitted for simplicity): The input is first fed into a convolutional layer (7 × 7 kernel-size, stride = 2, channel-number = 64) followed by a max-pooling (kernel-size = 3 × 3, stride = 2) layer; subsequently, 8 residual blocks are applied with each containing 2 convolutional layers. Residual blocks parameters: conv layers in block 1, 2 have kernel-size = 3 × 3 and channel = 64; conv layers in block 3, 4 have kernel-size = 3 × 3 and channel = 128; conv layers in block 5, 6 have kernel-size = 3 × 3 and channel = 256; conv layers in block 7, 8 have kernel-size = 3 × 3 and channel = 512; stride = 2 is applied at the first conv layer of block 3, 5, 7. Global average pooling is applied at the end. The classification head is made with one fully-connected layer.

In this work, we use a ResNet18^47^ as a 2D CNN backbone for feature extraction. Here, the 2D network essentially receives input from a small 3-slice subvolume as ResNet18 is designed to receive color images with 3 channels (RGB). We extract features from every 3 consecutive slices and combine features from all non-overlapping 3-slice subvolumes for the prediction head for classification (Fig. 1). Specifically, given input volumetric data with the shape of $S\hspace{0.17em}\times \hspace{0.17em}H\hspace{0.17em}\times \hspace{0.17em}W$ (S: slice number, H × W: slice size, with each slice in sagittal view), we generate $S$/3 2D 3-channel images each with the shape of $3\hspace{0.17em}\times \hspace{0.17em}H\hspace{0.17em}\times \hspace{0.17em}W$. The same ResNet18 is applied to extract features from each 3-slice subvolume and features from all $S$/3 3-slice subvolumes are concatenated as the input to a linear prediction head. The ResNet18 architecture is shown at the bottom of Fig. 1. The input is fed to a convolutional layer (conv layer) (kernel-size = 7 × 7, stride = 2, channel-number or number-of-feature-maps = 64), followed by a max-pooling layer for further downsampling (kernel-size = 3 × 3, stride = 2). After the pooling, 8 convolutional layer blocks called residual blocks (where input to the block is added to the output via residual short-cut connection) are applied where each block contains 2 convolutional layers with kernel-size = 3 × 3, the channel number gets doubled and the spatial size gets downsampled by 2 at the first conv layers of 3rd, 5th, 7th residual blocks. Each conv layer is followed by batch-normalization^55^ and ReLU activation^56^. In the end of ResNet18, global-average pooling is applied to each feature map to generate a single feature value, leading to 512 features for each 3-slice subvolume. Given $SxHxW$=180 × 224 × 224, we have $S$/3 = 60 3-slice subvolumes with each yielding 512 features. These 60 × 512 features are concatenated and fed to a linear layer for final prediction, which is a fully-connected layer mapping 60 × 512 features to the predicted class score.

#### 3D convolutional neural network

**Figure 2:**
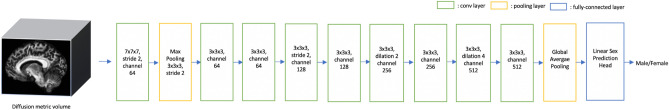
Our 3D CNN model based on ResNet10 (residual connection, ReLU activation, group normalization omitted for simplicity). The 3D volume is first fed to a conv layer (kernel-size = 7 × 7 × 7 stride = 2, channel = 64) followed by a max pooling (kernel-size = 3 × 3 × 3, stride = 2). Subsequently, 8 residual blocks are applied with each containing 1 conv layer. Residual blocks parameters: block 1, 2 have kernel-size = 3 × 3 × 3 and channel = 64; block 3, 4 have kernel-size = 3 × 3 × 3 and channel = 128; block 5, 6 have kernel-size = 3 × 3 × 3 and channel = 256; block 7, 8 have kernel-size = 3 × 3 × 3 and channel = 512; stride = 2 is used at conv layer in block 3, while dilation = 2 is used at conv layer in block 5 and dilation = 4 is used at conv layer in block 7. Global average pooling is applied at the end. The classification head is made with one fully-connected layer.

We employ 3D ResNet-10^48–50^ as our 3D CNN backbone, with architecture shown in Fig. 2. The 3D volume is firstly fed into a conv layer (kernel-size = 7 × 7 × 7, stride = 2, channel = 64) followed by a max pooling layer (kernel-size = 3 × 3 × 3, stride = 2), 8 residual blocks are then used with each block having 1 conv layer. The channel number is doubled at residual block 3, 5, 7, with stride set as 2 for block 3 and dilation set as 2 for block 5 and set as 4 for block 7. Each conv layer is followed by group-normalization^57^ and ReLU activation^56^. In the end, global average pooling is applied to map 512 feature maps to 512 feature values and one linear layer is used for the final prediction, which is a fully-connected layer mapping 512 features to the predicted class score.

#### Vision transformer for 3D input pretrained with mask autoencoders

**Figure 3:**
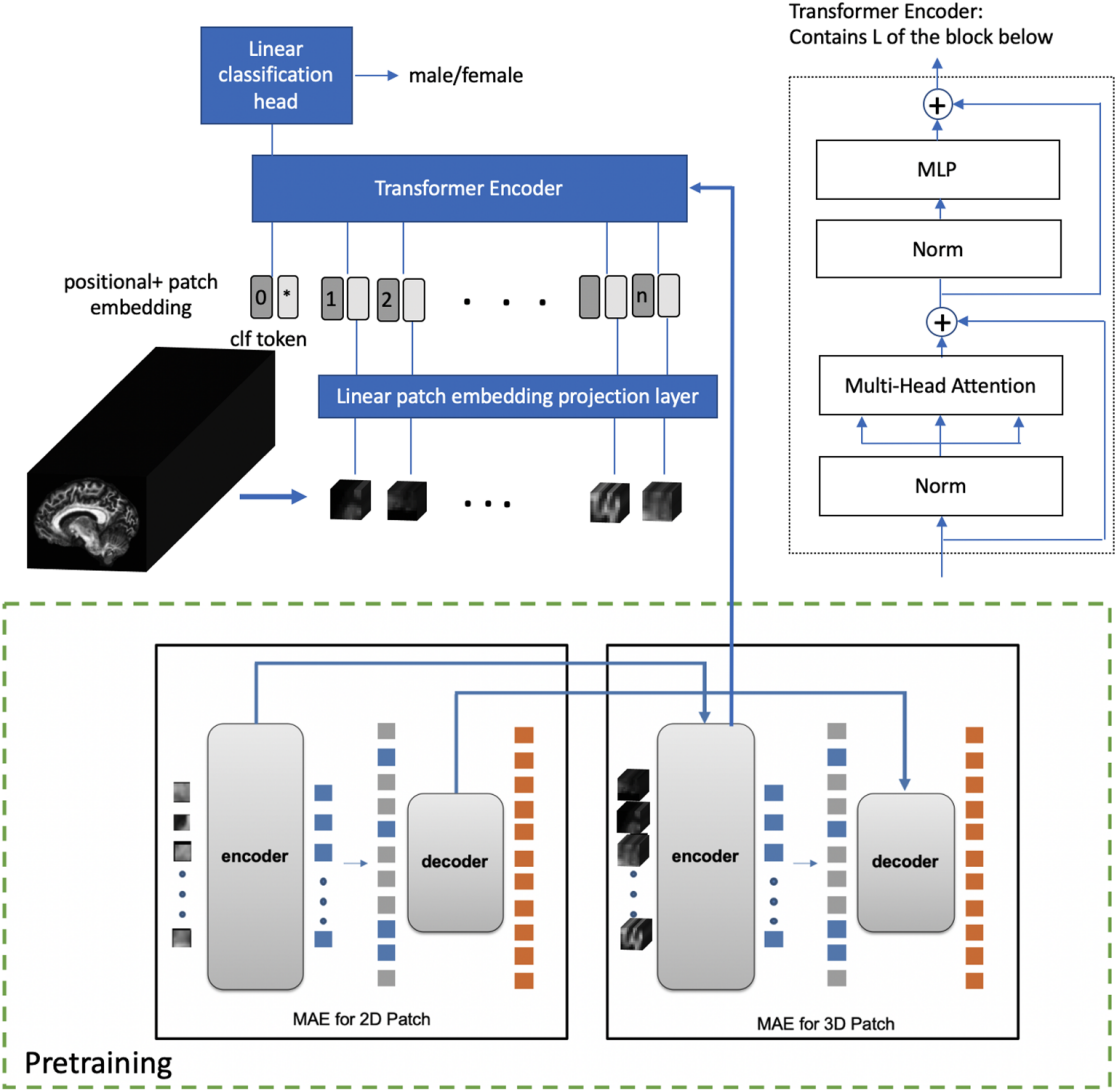
Vision Transformer for Diffusion MRI sex classification: the imaging volume inputted is partitioned into non-overlapping patches. Each patch is projected to patch embedding using a linear patch embedding layer, and added with positional embedding representing the position of the patch. A classification token is appended to the sequence of tokens to learn representation of the entire input sample. The structure of the transformer encoder is shown on the right, which consists of L alternating layers of multi-head attention and multiple-linear-perceptron (MLP) blocks. After the transformer encoder, the corresponding output of the classification token is fed to the classification head to generate prediction results. In our study, a 1 fully-connected layer is used as the classification head. The pretraining of the ViT is shown in the lower part of the figure, with details of the pretraining included in the supplementary information. The ViT encoder from the pretraining is used as the Transformer Encoder as the backbone for the sex classifier shown in the upper part of the figure.

The original 2D ViT^51^ is extended to extract features from a 3D volume. Given input 3D diffusion metric $x \in {R }^{S \times H \times W}$, the data is reshaped into a sequence of flattened non-overlapping 3D patches ${x}_{p} \in {R }^{N \times (s \cdot h \cdot w)}$, where ($S, H, W$) is 3D volume size and ($s, h, w$) is the 3D patch size, patch number is defined as $N = SHW/shw$. As shown in Fig. 3, for each 3D patch, a linear layer is applied to map voxel values to a latent embedding with dimension $D$. A learnable positional embedding with same dimension $D$ representing each token’s location, is added to the original embedding. The resulting sequence of embeddings for all N patches are fed to the encoder consisting of L alternating layers of multi-head attention and Multi-layer-perceptron (MLP) blocks. A classification token with dimension D is appended to the input embedding sequence, which is designed as a latent representing the entire input sample.The output embedding of the classification token is then fed into a linear prediction head to generate a prediction. In our study, $S\times H\times W=$ $182\times 224\times 224$ and $s\times h\times w=6\times 16\times 16$, $D=384$, $L=12$, and the classification head is a fully-connected layer mapping 384 to the predicted class score.

We pretrain the ViT with a 2D + 3D Masked Autoencoders (MAE) modified from 2D MAE^58^, where a specific ratio of patches, defined as $r$, is randomly masked and a ViT encoder and auxiliary decoder are trained to predict the values of $r \times N$ masked patches from $(1 - r)\times N$ unmasked patches. After pretraining, the encoder is finetuned for the target sex classification task with all $N$ patches fed into it. Since 3D patches are more difficult to predict than 2D patches (especially given the small number of available 3D volumes), we pretrain a 2D ViT encoder with MAE on 2D slices first and use the resulting weights to initialize our 3D ViT model for 3D patches, and further pretrain the model with MAE on 3D volumes. In our study, mask ratio $r = 0.75$ and the axillary decoder has $D=192$, $L=4$. Details of the pretraining can be found in the supplementary information.

### Model training and evaluation

1031 unique subjects are split into training (831 subjects), validation (100 subjects) and test sets (100 subjects). Training, validation and test sets share the same sex and age distribution, where female and male have a relatively balanced ratio of 27:23. Models’ hyperparameters are tuned based on the performance on the validation set. Models are then trained with the training set and tested on the test set for final prediction results. Classifiers are implemented with pytorch. For fair comparison, all three models use the same training/validation/testing split. Details of the training process are explained in the supplementary information. For ViT, we conducted three experiments: ViT trained from scratch without MAE pretraining, linear probing where the encoder is freezed with weights from MAE pretraining, and only linear prediction head is trained for sex classification, and fine-tuned ViT where the whole model is refined on sex labels. The performance of linear probing can reflect how the feature learnt from pretraining generalizes to the sex classification task, while performance of model trained from scratch can serve as the baseline to examine if the pertaining can bring performance improvement. Details of the training are described in supplementary information.

### Occlusion analysis and Wilcoxon signed rank test

We conduct occlusion analysis on the trained models and Wilcoxon signed rank test to identify white matter areas of the brain that contribute significantly to sex classification. We conduct occlusion at the region level and consider the 48 white matter regions defined by the Johns Hopkins University-ICBM-labels-1 mm atlas^45^. Given a trained model for a diffusion metric, we compare the predicted probability for the correct label before and after occlusion of each region in succession, by setting all voxels in the region to the mean white matter value. We apply the Wilcoxon signed rank test with the one-sided alternative hypothesis to the probability changes associated with each region for all subjects in the testing dataset to test whether the decrease in the predicted probability for the correct label is statistically significant. The regions that achieve p-value < 0.05 are considered significant for distinguishing between male and female.

### Ethics approval

This study was conducted in compliance with the Health Insurance Portability and Accountability Act and approved by the institutional review board.

## Result

### Classification results

We use the area under the curve (AUC) of each trained model on the testing dataset to evaluate the model performance. Besides, accuracy, precision and recall are also included. Table 2 shows that our 2D CNN, 3D CNN and ViT (fineturned and linear probing) models all achieved promising AUC for all 3 diffusion metrics with test AUC of > 0.9. For FA and MD, 2D CNN achieved the highest AUC at 0.98 for FA and at 0.97 for MD. 3D CNN and ViT also achieved relatively high AUC (> 0.92). For MK, all models achieved a high AUC above 0.96, and 3D CNN achieved highest performance with AUC of 0.98. The ViT trained from scratch yielded low AUC (< 0.8) for all diffusion metrics. The finetuned ViT and linear probing ViT achieved comparable AUC on all 3 diffusion metrics, indicating that the MAE-pretrained feature extraction layer is directly applicable for the classification task.

**Table 2:** Performance (test AUC**/**Accuracy/Precision/Recall) of sex classification models using three different diffusion MRI parametric maps as inputs (FA, MD, and MK).

Model	FA	MD	MK
2D CNN	0.98/0.91/0.93/0.87	0.97/0.90/0.87/0.91	0.96/0.89/0.89/0.87
3D CNN	0.92/0.87/0.88/0.82	0.96/0.86/0.84/0.84	0.98/0.88/0.97/0.76
ViT (finetuned)	0.93/0.83/0.80/0.82	0.95/0.87/0.83/0.89	0.97/0.89/0.93/0.82
ViT (linear probing)	0.94/0.87/0.81/0.93	0.95/0.87/0.85/0.87	0.96/0.90/0.89/0.89
ViT (trained from scratch)	0.79/0.69/0.72/0.51	0.75/0.66/0.62/0.62	0.72/0.66/0.60/0.73

### Occlusion analysis

**Figure 4:**
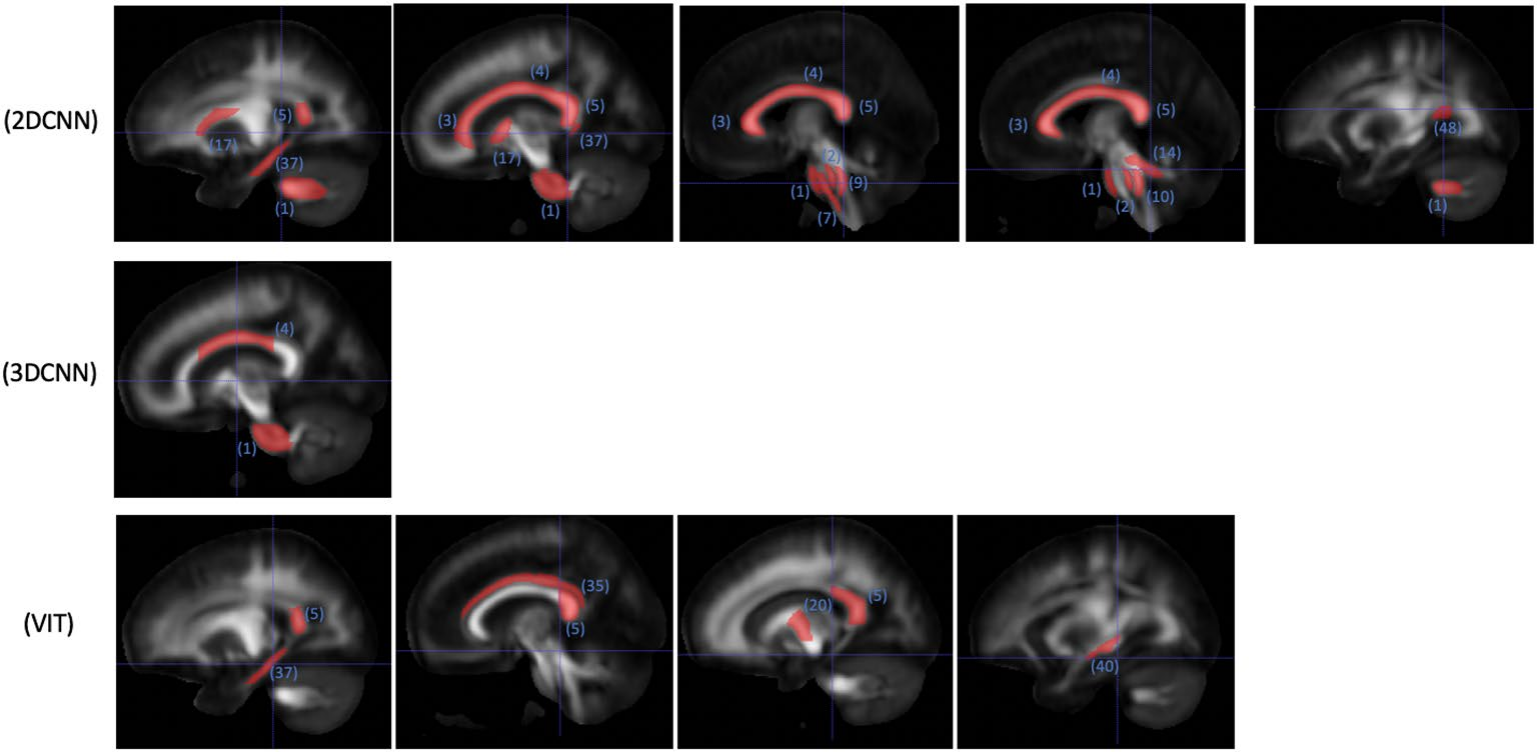
WM regions with significant (p < 0.05) impact on classification probability based on occlusion analysis for FA; numbered labels based on JHU-ICBM-1 mm atlas (https://identifiers.org/neurovault.image:1401); 1: middle cerebellar peduncle, 2: pontine crossing tract (a part of middle cerebellar peduncle), 3: genu of corpus callosum, 4: body of corpus callosum, 5: splenium of corpus callosum, 7: corticospinal tract (right), 9: medial lemniscus (right), 10: medial lemniscus (left), 14: superior cerebellar peduncle (left); 17: anterior limb of internal capsule (right), 20: posterior limb of internal capsule (left), 35: cingulum (cingulate gyrus) (right), 37: cingulum (hippocampus) (right), 40: stria terminalis (left), 48: tapetum (left).

**Figure 5:**
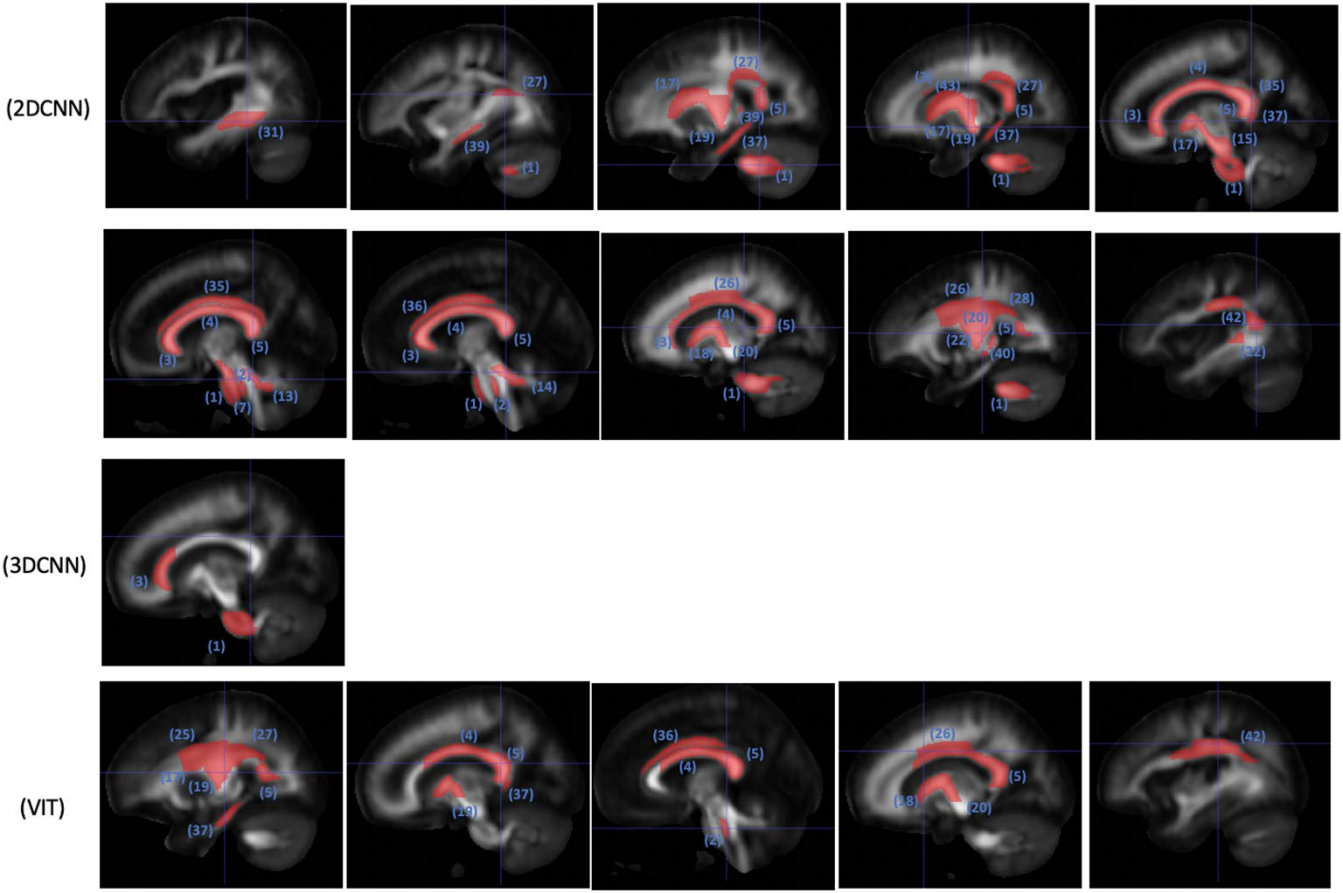
WM regions in selected slices with significant (p < 0.05) impact on classification probability based on occlusion analysis for MD; numbered labels based on JHU-ICBM-1 mm atlas (https://identifiers.org/neurovault.image:1401); 1: middle cerebellar peduncle, 2: pontine crossing tract (a part of middle cerebellar peduncle), 3: genu of corpus callosum, 4: body of corpus callosum, 5: splenium of corpus callosum, 5: splenium of corpus callosum, 7: corticospinal tract (right), 13: superior cerebellar peduncle (right), 14: superior cerebellar peduncle (left), 15: cerebral peduncle (right), 17: anterior limb of internal capsule (right), 18: anterior limb of internal capsule (left), 19: posterior limb of internal capsule (right), 20: posterior limb of internal capsule (left), 22: retrolenticular part of internal capsule (left), 25: superior corona radiata (right), 26: superior corona radiata (left), 27: posterior corona radiata (right), 28: posterior corona radiata (left), 31: sagittal stratum (right), 35: Cingulum (cingulate gyrus) (right), 36: cingulum (cingulate gyrus) (left), 37: cingulum (hippocampus) (right), 39: stria terminalis (right), 40: Stria terminalis (left), 42: superior longitudinal fasciculus (left), 43: superior fronto-occipital fasciculus (right).

**Figure 6:**
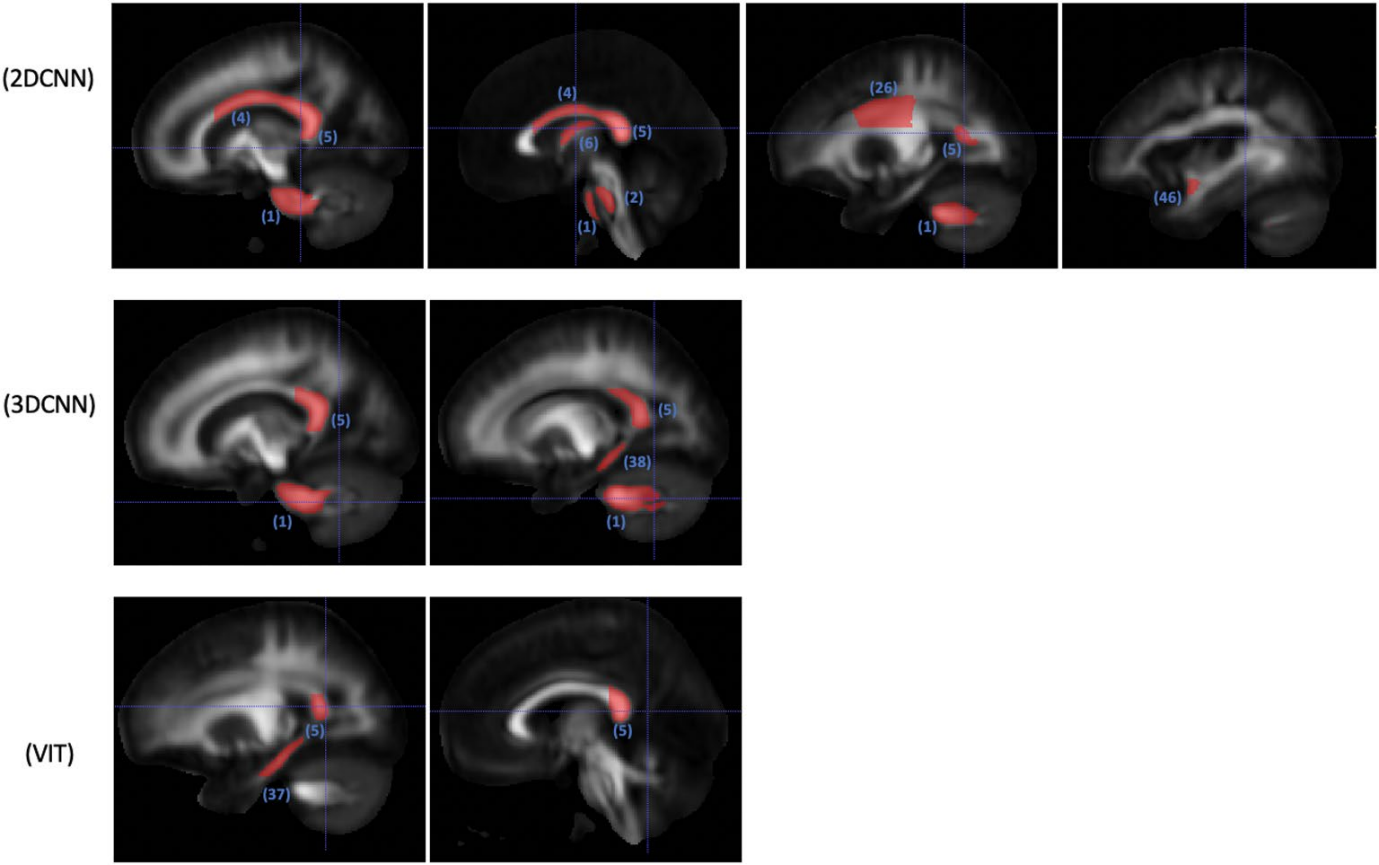
WM regions in selected slices with significant (p < 0.05) impact on classification probability based on occlusion analysis for MK; numbered labels based on JHU-ICBM-1 mm atlas (https://identifiers.org/neurovault.image:1401); 1: middle cerebellar peduncle, 2: pontine crossing tract (a part of middle cerebellar peduncle), 4: body of corpus callosum, 5: splenium of corpus callosum, 6: fornix (column and body of fornix), 26: superior corona radiata (left), 37: Cingulum (hippocampus) (right), 38: cingulum (hippocampus) (left), 46: uncinate fasciculus (left).

2D and 3D CNNs and finetuned ViT are included in the occlusion analysis. The ViT finetuned model is selected for the occlusion analysis despite it has similar performance as the linear probing model, because the finetuned model is refined on the sex classification task. The numbers of regions passing the significance test are summarized in Table 3. Identified regions are illustrated in Figs. 4, 5, and 6.

**Table 3:** Number of white matter regions showing significant differences between males and females in the occlusion analysis; 48 WM regions in total.

Model	FA	MD	MK
2D CNN	12	25	7
3D CNN	2	2	3
ViT (finetuned)	5	13	2

## Discussion

The study provides new evidence of clear sex-related differences in white matter microstructure as captured by diffusion MRI, detected consistently across 3 different end-to-end, deep learning-based image classification models. The reliability of this finding is evident in the fact that high classification performance (test AUC 0.92–0.98) is observed independent of model architecture across 3 major network architecture types and without introducing the biases of complex hand-crafted features and/or manual operations as has been done previously. In addition, white matter regions most influential in model decision are identified and show the location and distribution of greatest microstructural differences.

Use of the three different model architectures intentionally allows us to leverage the different strengths of each of these network families. For example, the 3D CNN incorporates a conventional 3D CNN backbone: while it powerfully captures local features within the volume, a recent study showed that even very deep CNNs with a high number of parameters still have only small effective receptive fields^54^, meaning they rely primarily on their ability to learn local features as opposed to longer distance relationships. On the other hand, ViTs capture global features more readily^51^ and the incorporated MAE pretraining task used here also heavily focuses the model on inter-patch correlations than span some distance across the volume. The study finds that both the 3D CNN and ViT models performed very well, suggesting that there are both short-distance and long-distance interactions that show differences in terms of sex-related patterns of white matter microstructure.

The 2D CNN achieved overall best performance for 2 out of 3 diffusion metrics studied. This is felt to be attributable to the fact that the 2D CNN incorporates a design that may allow it to simultaneously capture both local features and global interactions (across all slices), thus making it able to leverage both types of features in the classification task. Specifically, the ResNet18 extracts features from every group of 3 consecutive slices allowing the model to learn from within-slice features and short-range inter-slice features across the 3 slices; by then concatenating features across all 3-slice subvolumes (as opposed to averaging across them as is most commonly done) the model here effectively preserves local features from every 3-slice partition while at the same time, the prediction head is able to learn more global interactions across 3-slice subvolumes. It’s also possible that the simplicity of the 2D CNN model (it had the lowest number of parameters, as shown in Table 4) helps to push its generalization capability; although differences between test performance were nominal across all three models, which suggest that they are all comparably generalizable.

**Table 4:** The parameter number of three model architecture.

	Feature extraction backbone	Classification head	Tool
2D CNN	11,176,512	30,721	11,207,233
3DCNN	14,355,520	1026	14,356,546
ViT	24,143,232	770	24,144,002

The occlusion results show general consistency across models and across diffusion metrics and implicate central white matter tracts and ventral/dorsal hindbrain tracts in contributing to sex-related differences, though results differ slightly across diffusion metrics and models. The number and fractional volume of WM regions significantly contributing to sex classification was highest for 2D CNN (mean number of regions: 15; mean fractional volume: 0.79) compared with 3D CNN (mean number of regions: 2; mean fractional volume: 0.24) and ViT (mean number of regions: 7; mean fractional volume: 0.16), possibly again reflecting differences in the relative facility of these models to tap short-range interactions, long-range interactions, or both. Across the three diffusion metrics, it appears that the 3D CNN classifier focused consistently on large central white matter structures such as the middle cerebellar peduncle and corpus callosum whereas the ViT and 2D CNN models tended to rely on a greater diversity of white matter regions. Another observation is that the corpus callosum was found to be important across all neural network architecture types and diffusion metrics. As sex-related regional brain structure differences have been particularly controversial^17–19^, our work provides new evidence that sex differences do in fact exist in focal regions such as the corpus callosum.

Of note for the ViT, pre-training with MAE was important. ViT is a data-hungry architecture and difficult to train with a limited dataset since it lacks inductive bias such as the locality and translation invariance of CNNs. The MAE pretraining task (to predict masked patches from visible patches) enables the model to learn inter-patch interactions without supervision from data labels. The random masking itself also introduces data diversity to the pretraining, which helps further improve the generalizability of learned features. The benefit of MAE pretraining is clearly demonstrated in the experimental results: without pretraining, ViT trained from scratch yielded much lower performance with test AUC < 0.80. The improvement of each of the other models compared to ViT trained from scratch is statistically significant, with *p* < 0.05 achieved with the Wilcoxon signed-rank test that compares the predicted probability of the correct class. With MAE pretraining, the ViT encoder achieved test AUC 0.94–0.96. The end-to-end supervised finetuning brought no additional gain and achieved comparable performance with linear probing, confirming that the size of the training set is insufficient to tune a data-hungry ViT in supervised end-to-end training.

Limitations include the use of only three representative diffusion metrics, though these are chosen based on the fact that they are the most common and easily obtained using a well-established diffusion kurtosis imaging acquisition. Further exploration using modeled diffusion metrics^26^ may yield additional information about sex-related differences in tissue microstructure and help us continue to characterize the underlying biophysical differences between brains of males and females. Additionally, our study is based on DWI with moderate b-value, future study can include datasets with high b-value that are more sensitive to restricted diffusion^59^. Recognizing that the age distribution differs between the female and male cohorts (with the female group having more older people) (Table 1), we have separately evaluated the model accuracy on three subgroups broken down by age and found the performance to be comparable across all age groups. For a narrow-band of adults ages 26–30 where aging changes are likely to have little influence, our models continue to achieve high sex classification accuracy. In future work, combined with additional diffusion metrics such as the modeled diffusion metrics^26^, the study can be extended to examine the sex differences in age ranges other than young adults, which can shed light on how sex differences progress in life span. Finally, occlusion analysis used the standard JHU-ICBM-1 mm atlas for white matter parcellation which has sizable variation in size of regions which could potentially bias regional importance; any affect region size has on region importance is limited, however, as the results for relative importance of regions does not correlate well with region size. Besides, our 2D CNN is based on sagittal slices, in future work, the new 2D CNN can be designed to leverage information from all three views. This study does not use the 3 diffusion metrics as combined input to neural networks due to GPU memory constraint; neural network architectures that can efficiently take multiple 3D volumes as combined input can be explored in the future work.

Overall, our results provide new evidence and insights to support that sex differences exist in human brain microstructure both in local features (e.g., within central white matter structures such as the middle cerebellar peduncle and corpus callosum) and in global features (like long-distance interactions). Capturing complex microstructural differences is challenging using conventional statistical methods or single-approach machine learning network inputting handcrafted features. Our work demonstrates a unique approach: that by leveraging multiple neural networks with completely different architecture design, it allows us to capture complementary information and makes our results independent of model architecture choices. When it comes to using advanced machine learning architectures that are data-hungry, self-supervised learning can be used to pretrain models and enable such neural networks to be leveraged for medical imaging studies that lack tremendous datasets such as those available for non-medical, computer vision work. Such a framework can be further adapted to study other neurological disorders.

## Conclusion

This study provides new evidence of clear sex-related differences in brain white matter microstructure of healthy young adults detected using in vivo diffusion MRI without hand-crafting or manually manipulating the imaging data. We show this across 3 different end-to-end deep neural networks and 3 commonly used diffusion MRI metrics. Even after registering diffusion MR volumes to a template so as to remove macroscopic anatomical differences such as overall brain size and contour, results show that sex differences exist in diffusion anisotropy (FA), mean diffusivity (MD) and tissue complexity (MK) of brain white matter. Our experiments further suggest there are both local as well as longer-distance microstructural organizational features that differ between sexes. In particular, the central white matter appears specifically implicated. In addition, this study provides a framework to study microstructural differences in the human brain using multiple deep neural network architectures, which help capture complex microscopic features challenging for statistical methods. Further study is needed to determine whether and how these microstructural differences influence brain health and disease in both men and women.

### Supplementary Information

Supplementary Information.

## Data Availability

The dataset used in this study can be downloaded from the Human Connectome Project via Amazon S3 at https://db.humanconnectome.org/.
