# Author Correction: Deep learning with diffusion MRI as in vivo microscope reveals sex-related differences in human white matter microstructure

**DOI:** 10.1038/s41598-024-66995-x

**Published:** 2024-08-05

**Authors:** Junbo Chen, Vara Lakshmi Bayanagari, Sohae Chung, Yao Wang, Yvonne W. Lui

**Affiliations:** 1 Department of Electrical and Computer Engineering, New York University Tandon School of Engineering, 370 Jay Street, 9th Floor, Brooklyn, NY 11201 USA; 2 Center for Advanced Imaging Innovation and Research (CAI2R), Department of Radiology, New York University Grossman School of Medicine, New York, NY USA; 3 Bernard and Irene Schwartz Center for Biomedical Imaging, Department of Radiology, New York University Grossman School of Medicine, New York, NY USA; 4 Department of Biomedical Engineering, New York University Tandon School of Engineering, Brooklyn, NY USA

Correction to: *Scientific Reports* 10.1038/s41598-024-60340-y, published online 14 May 2024

In the original version of this Article, an incorrect citation was included.

“Zhang, H., Schneider, T., Wheeler-Kingshott, C. A. & Alexander, D. C. NODDI: practical in vivo neurite orientation dispersion and density imaging of the human brain. *Neuroimage.* **61**(4), 1000–1016 (2012).”

As a result, in the Discussion section,

“Further exploration using modeled diffusion metrics^26^ and neurite orientation dispersion and density imaging (NODDI)^60^ may yield additional information about sex-related differences in tissue microstructure and help us continue to characterize the underlying biophysical differences between brains of males and females.”

now reads:

“Further exploration using modeled diffusion metrics^26^ may yield additional information about sex-related differences in tissue microstructure and help us continue to characterize the underlying biophysical differences between brains of males and females.”

“In future work, combined with additional diffusion metrics such as the modeled diffusion metrics^26^ and NODDI^60^, the study can be extended to examine the sex differences in age ranges other than young adults, which can shed light on how sex differences progress in life span.”

now reads:

“In future work, combined with additional diffusion metrics such as the modeled diffusion metrics^26^, the study can be extended to examine the sex differences in age ranges other than young adults, which can shed light on how sex differences progress in life span.”

The original Article has been corrected.

