# Supplementary material for: Deep learning with diffusion MRI as in vivo microscope reveals sex-related differences in human white matter microstructure
Source: Sci Rep. 2024 May 14;14:9835. doi: 10.1038/s41598-024-60340-y (PMC11094063; doi:10.1038/s41598-024-60340-y)
Supplement: Supplementary file 1 — Supplementary Information. [file 41598_2024_60340_MOESM1_ESM.docx]

**Deep Learning with Diffusion MRI as in vivo Microscope Reveals Sex-related Differences in Human White Matter Microstructure**

|  |
| --- |

**Appendix:**

*Vision Transformer: 2D+3D MAE Pretraining and Model Architecture*

We apply a 2D+3D MAE for the ViT pretraining. In MAE based pretraining, a specific proportion of patches (defined as masking ratio $r$) are randomly masked out and the model is trained to inpaint the masked patches with the remaining visible patches. By training the model to do this mask autoencoding pretask, the model is expected to learn features that are generalizable to various target downstream tasks. Specifically, with $r$ as mask ratio and $N$ as patch number, the $(1 - r)\cdot N$ visible patches are fed to a ViT encoder to extract latent features, then $r\cdot N$ learnable mask tokens are inserted back to the latent sequence and fed to a transformer decoder. The final latents of masked patches then go through a prediction head to predict each masked patch’s pixel/voxel values. Following ViT and MAE implementations^47,57^, a classification token $[class]$ is appended to the token sequence. After MAE pretraining, the decoder is removed and the encoder is used to extract latent features from the input volume where all patches are visible. The classification prediction head is attached to the $[class]$ for the downstream classification task.


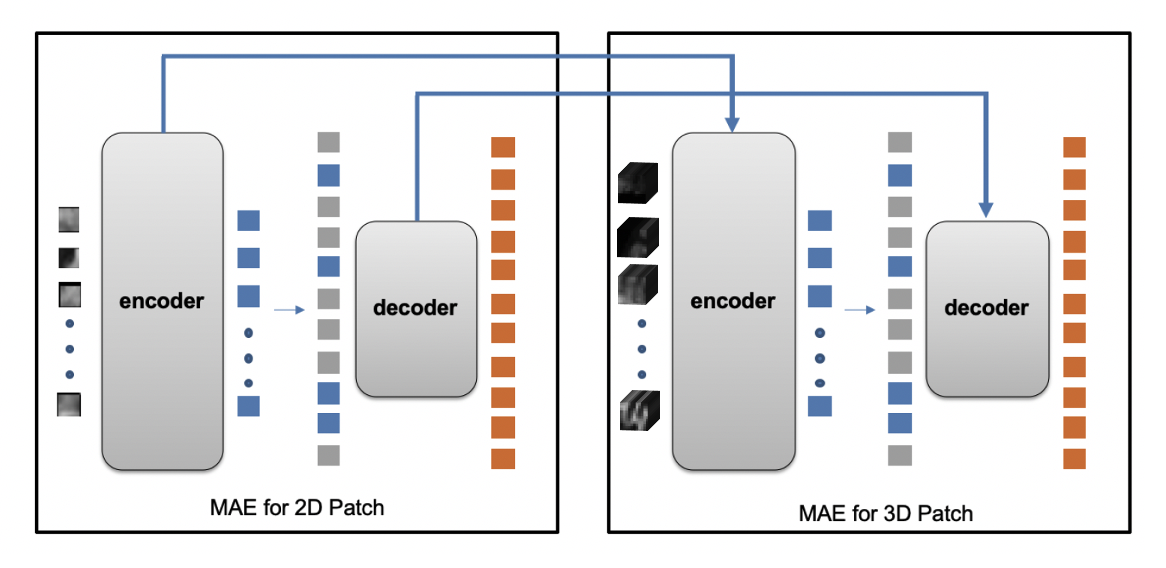


Fig A.1 Transformers in 3D MAE initialized with 2D MAE

Compared with 2D patches, 3D patches are more difficult to predict especially when only limited 3D volumes are available. Therefore, a 2D+3D MAE is applied in our study. Instead of directly training MAE on 3D volumes, we first treat each slice as an individual sample and train encoder and decoder to do MAE on 2D slices to learn intra-slice correlation between patches. The the model pretrained with 2D MAE (with learned intra-slice interaction) is then used to initialize the encoder and decoder for MAE on 3D volumes that aims to further learn inter-slice interaction, showed in the Figure A.1. There are only 3 blocks that are different between MAE for 2D slices and MAE for 3D volumes: linear layer in the encoder to generate patch embeddings, linear layer in decoder to predict patch pixels/voxels, and positional embeddings. Inspired by Video Swin-Transformer^58^, we inflate the weights of these 2 linear layers and positional embeddings of 2D MAE by $s$ to match the shapes of weights in 3D MAE. For encoder, the patch embedding layer for 2D patches has the weight size of $(hwc) \times(D)$, the weight is then duplicated $s$ time as $(shwc) \times(D)$ for 3d patches. The same idea is applied to the linear pixels/voxels prediction head in the decoder and positional embeddings.

The architecture of ViT encoder and decoder (decoder is only used in the MAE pretraining) is shown in Table A.1. The encoder and decoder adapt the asymmetric architecture following the MAE designed for images^57^, as encoder only operates on visible patches and decoder operates on all patches during the pretraining, decoder is more memory consuming and should employ a smaller architecture. For the input metric, the axial view slice is interpolated to $HxW=$ 224 x 224, with $S=182$. The patch size is set as $s x h x w=$ 6 x 16 x 16 (transverse plane: $h x w=$ 16x16; sagittal plane:$s x h$ = 16x6), As diffusion MRI is relatively monotonous within white matter tracts, the patch size is tuned to be just large enough to make most patches contain meaningful structure of white matter tracts. The masking ratio $r$ is set as 75% as it was shown to be the best ratio for the image data, making the MAE task both feasible and challenging enough to learn generalizable features^57^.

|  | Encoder | Decoder |
| --- | --- | --- |
| Latent Dimension | 384 | 192 |
| Depth | 12 | 4 |
| Number of Heads | 6 | 3 |
| MLP Ratio | 4 | 4 |

**Table A.1** The architecture of ViT encoder and decoder

*Model Training and Evaluation*

For ViT, the model is first pretrained with 2D MAE on slices of axial views. Then the model is then initialized with weights from 2D MAE and pretrained one 3D MAE. After pretraining, the encoder is used to extract features for sex classification. To evaluate if the learned features from the MAE pretraining is generalizable to the sex classification downstream task, we conduct a linear probing experiment that is commonly used to evaluate self-supervised learning methods: we freeze the ViT encoder and only train a linear classifier on sex labels, and evaluate the classification AUC achieved by the linear classifier and compare it with ViT trained from the scratch. Given good linear probing results, we then finetuned the encoder and classification layer in an end-to-end manner on sex labels to get the final ViT models for sex classification. For MAE, the model is trained with the mean square error between predicted pixels/voxels and their reconstruction target ground truth value for all masked patches. Instead of using the patches’ original value, the reconstruction target is set as the values after z-score normalization with the mean and standard deviation of pixels/voxels in the patch, as this normalized target can help improve the representation quality of the pretraining^57^. The AdamW optimizer is used with $\beta_{1}=-0.9$and $\beta_{2}=0.95$. The weight decay is set as 0.05. For the 2D MAE, the mode is trained for 500 epochs with batch size as 128 and initial learning rate as $7.5 x {10}^{-5}$. For the 3D MAE, the model is trained for 600 epochs with batch size as 8 and initial learning rate as $4.5 x {10}^{-6}$. For linear probing, logistic regression models from Scikit-learn^59^ are trained on latents from frozen pretrained ViT encoder. For end-to-end finetuning, the model is trained for 100 epochs with initial learning rate as $1 x {10}^{-4}$ and batch size 2 with cross-entropy as the loss function.

For 3D CNN, the stochastic gradient descent optimizer is used with momentum as 0.9 and weight decay as 0.001. Exponential learning rate scheduler with $\gamma=0.99$ is used. The model is trained for 100 epochs with initial learning rate as 0.01 and batch size 8.

For 2D CNN, Adam optimizer is applied with learning rate at 0.03, momentum as 0.9 and beta values $\beta_{1}=-0.9$and $\beta_{2}=0.999$. The model was trained for 50 epochs with batch size set as 10. Given the higher number of female samples than male samples, we found the 2D CNN prediction is biased toward females and solved it by weighting cross-entropy loss with class-weights (males=0.45 and females=0.55).

*Occlusion Analysis: 3D visualization of regions with significance*


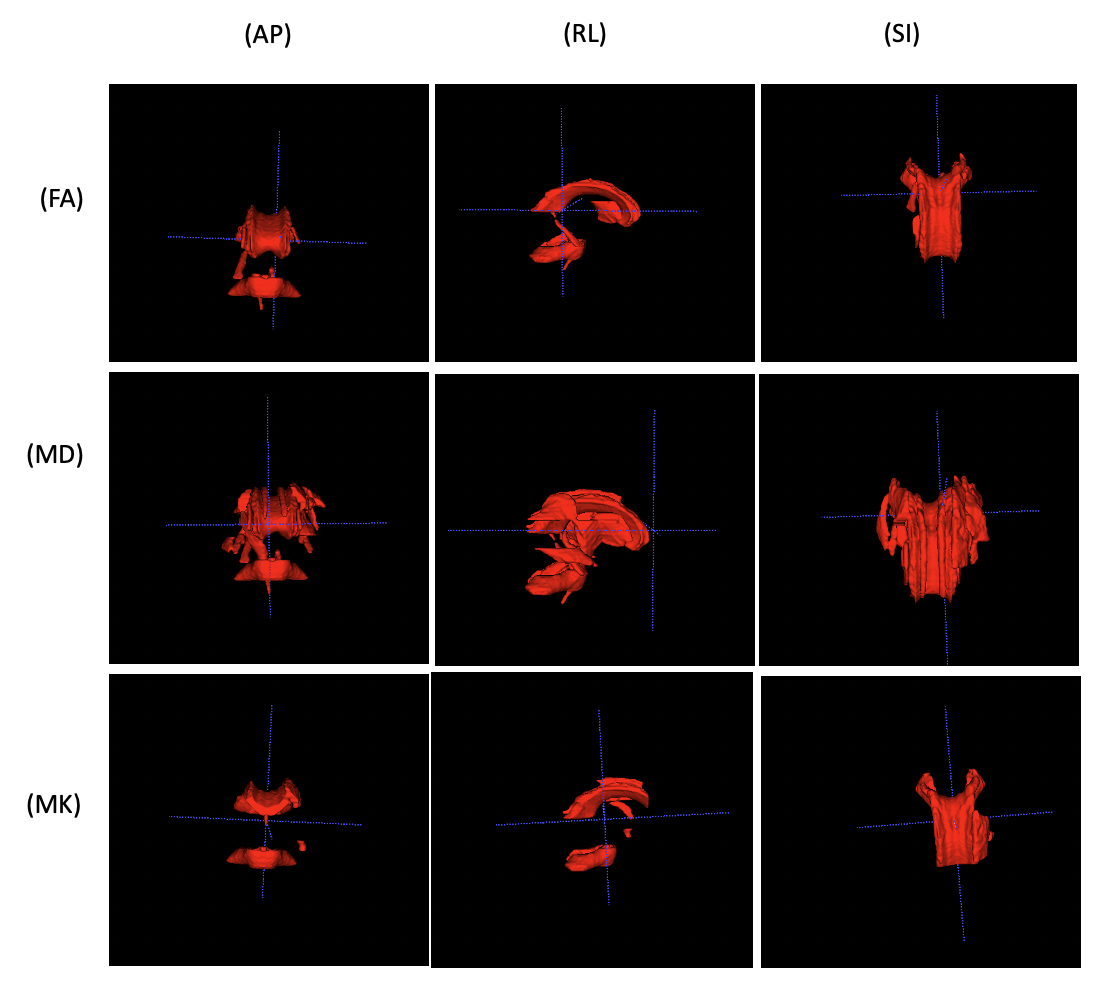


Fig A.2 3D visualization of wm regions with significance for 2D CNN; AP: anterior to posterior; RL: right to left; SI: superior to inferior.


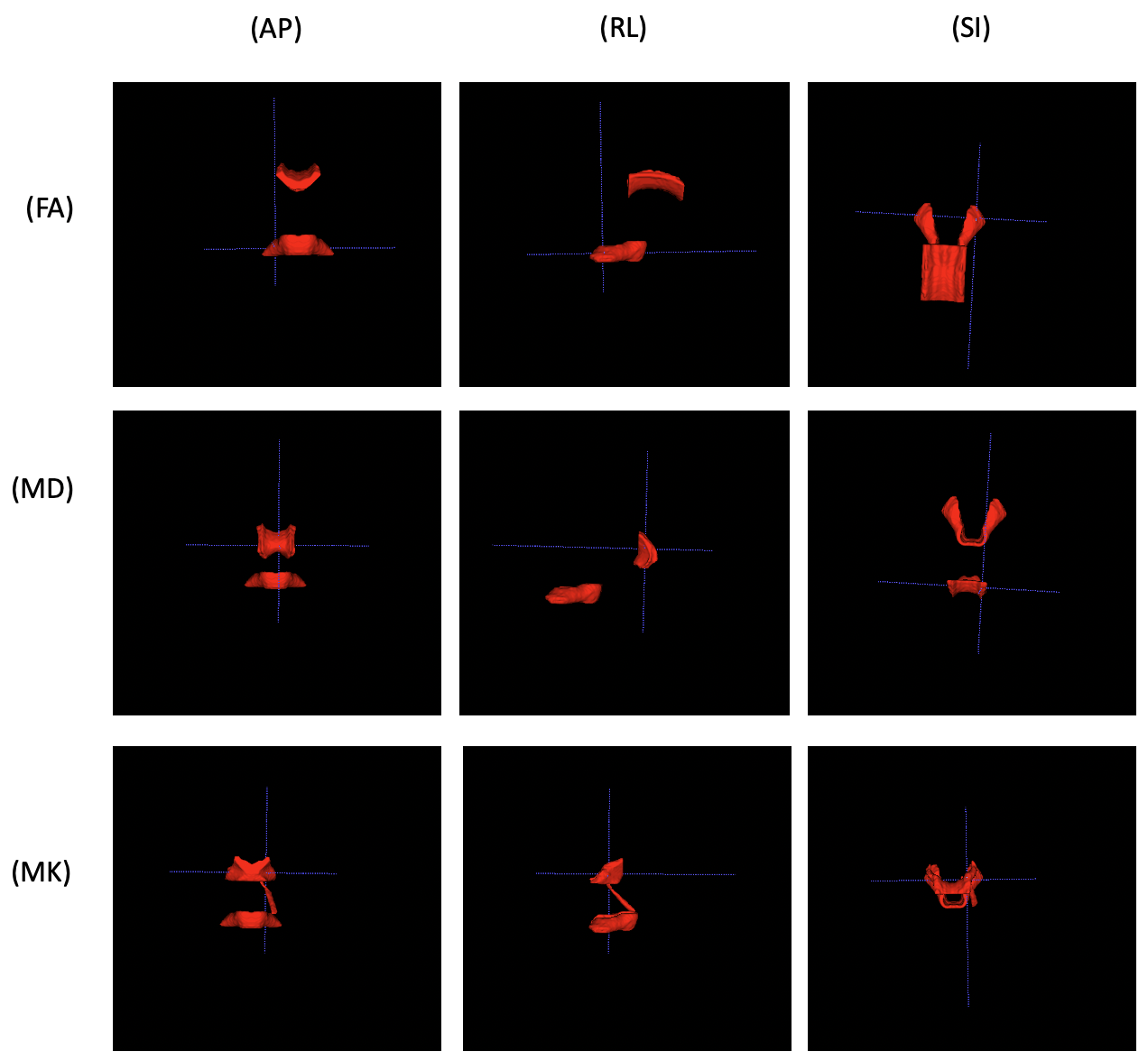


Fig A.3 3D visualization of wm regions with significance for 3D CNN; AP: anterior to posterior; RL: right to left; SI: superior to inferior.

*
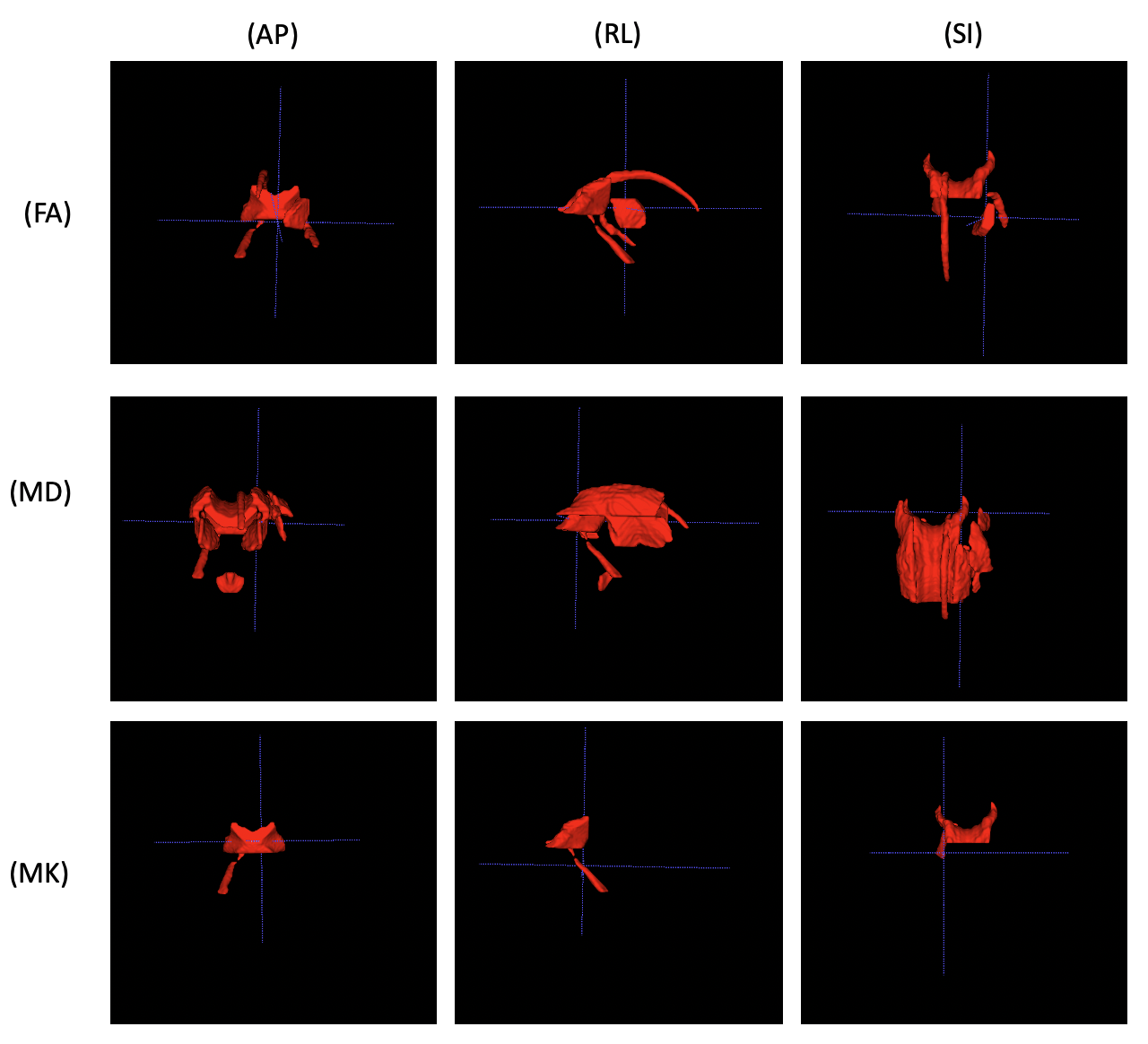
*

Fig A.4 3D visualization of wm regions with significance for ViT; AP: anterior to posterior; RL: right to left; SI: superior to inferior.
